# Imaging findings in chikungunya fever

**DOI:** 10.1590/0100-3984.2017.50.2e1

**Published:** 2017

**Authors:** Clarissa Canella

**Affiliations:** 1 Adjunct Professor of Radiology at the Universidade Federal Fluminense (UFF), Niterói, RJ, MD, Radiologist, Specialist in Musculoskeletal Imaging at the Clínica de Diagnóstico por Imagem (CDPI), Rio de Janeiro, RJ, Brazil. E-mail: clacanella@yahoo.com.br

Chikungunya fever is an infection that manifests clinically as acute fever and skin rash, together with disabling arthralgia, arthritis, and fatigue. The disease is caused by a virus of the family Togaviridae. The virus is transmitted to humans by the mosquitoes *Aedes aegypti* and *Aedes albopictus*. The diagnosis of chikungunya fever is made primarily on the basis of the clinical profile. Biochemical confirmation is absolutely necessary in order to differentiate the symptoms and make the differential diagnosis with other diseases transmitted by *Aedes* mosquitoes, such as dengue, that are endemic to the same geographic areas. To make the definitive diagnosis of chikungunya fever, the presence of the virus in the blood should be demonstrated directly by means of viral RNA determination at the peak of viremia; that is, 5–10 days after the onset of symptoms^(1,2)^. There are currently few therapeutic options, and symptomatic treatment produces only a slow, moderate response^(1)^.

Although arthralgia is the most typical articular manifestation, arthritis with significant synovitis can be seen in all phases of the disease, as demonstrated on ultrasound by distension of articular recesses due to joint effusion and hypoechoic synovial thickening that was incompressible, resulting in articular capsule bulging and tendinous adjacent structures. In some cases, power Doppler reveals signs of synovial hypervascularization. The involvement is usually distal, symmetrical, and polyarticular, predominantly occurring in the hands, wrists, and ankles. More rarely, the disease affects the elbows, knees, shoulders, hips, and temporomandibular joints. Heel enthesitis and sternal involvement occur less commonly^(2)^.

In chikungunya fever, there is a high incidence of recurrence and chronicity of joint involvement, with persistence of the inflammatory symptoms^(1,2)^. In the chronic phase, the disease presents aspects quite similar to those of rheumatoid arthritis, including bilateral, symmetric chronic polyarthritis with a migratory pattern, and the prevalence of rheumatoid factor positivity ranges from 25% to 43%^(1,2)^. After the initial manifestations, there can be recurrence of the arthritis, the rate of such recurrence decreasing over time, from 88–100% in the first six weeks to 12% by five years. Some authors emphasize the need for rheumatology follow-up of patients with chronic arthralgia, in order to identify cases that could eventually evolve to secondary rheumatoid arthritis^(2)^.

Unlike some fungal infections that are quite common in our country, such as paracoccidioidomycosis, which has been the subject of several recent publications in the radiology literature of Brazil^(3-6)^, very little has been written about the imaging aspects of infections with viruses transmitted by the Aedes mosquito. Imaging methods such as ultrasound and magnetic resonance imaging could play a key role in documenting joint involvement in the acute phase of chikungunya fever, especially in patients who develop chronic arthritis^(2)^.

Another important manifestation described in chikungunya fever is tenosynovitis of the hands, wrists, and ankles, which can be serious, leading to carpal/tarsal tunnel syndrome. This alteration can be identified on ultrasound by the liquid distension and thickening of the synovial tendon sheath, and power Doppler imaging shows signs of hypervascularization around the tendon in some cases. Tendon involvement has been described in the literature and seen in clinical practice, as reported in the study conducted by Mogami et al.^(7)^ and published in this issue of **Radiologia Brasileira**, in which the authors describe the ultrasound aspects of ankle involvement in chikungunya fever. The authors found that the disease occurs predominantly in females, and the abnormalities most often seen on ultrasound were joint effusion and tenosynovitis, mainly of the fibulae and posterior tibiae. Myositis of the soleus or flexor hallucis longus muscle was observed in some patients, as was retrocalcaneal bursitis.

Although the chikungunya virus was initially isolated in Tanzania in 1952, chikungunya fever became statistically significant only in 2004, after an epidemic in Kenya. However, it is worth noting that articular manifestations such as arthritis and synovitis have been reported in the literature since 1980, when Kennedy et al.^(8)^ reported such findings in a study of 20 patients with chikungunya fever. Those authors also found that there was chronicity of the clinical profile, with symptoms persisting for more than four months.

There was a significant increase in the number of cases of chikungunya fever in Brazil in 2016, predominantly in the northeastern region. According to Mogami et al.^(7)^, Brazilian health authorities expect an even greater increase in 2017.
